# Systematic review and meta-analysis of refractive errors in Ghana with emphasis on myopia

**DOI:** 10.1186/s12886-026-05179-9

**Published:** 2026-08-03

**Authors:** Sylvester Kyeremeh, Khathutshelo Percy Mashige, Kovin Shunmugam Naidoo, Kingsley Emwinyore Agho

**Affiliations:** 1 Discipline of Optometry, University of KwaZulu-Natal, Westville Campus, Durban, 3629 South Africa; 2 School of Medicine, Western Sydney University, Campbelltown, NSW Australia

**Keywords:** Myopia, Refractive error, Cycloplegia, Fogging

## Abstract

**Background:**

Refractive errors remain the leading cause of visual impairment globally, with myopia representing the most prevalent. In Ghana, reported prevalence estimates for refractive errors and myopia show considerable variability across studies, which complicates accurate quantification of their public burden and poses challenges for evidence-informed policy formulation and planning. The study aimed to estimate the prevalence of refractive errors in Ghana, with particular emphasis on myopia.

**Method:**

The systematic review followed the 2020 Preferred Reporting Items for Systematic Reviews and Meta-Analysis (PRISMA) guidelines and was prospectively registered with the Prospective Register of Systematic Reviews (PROSPERO; registration number CRD42022377958). A comprehensive and independent search of the literature was undertaken to identify peer-reviewed cross-sectional studies, using predefined Medical Subject Heading (MeSH) and relevant keyword subheadings across five electronic databases: PubMed, ProQuest, Scopus, Google Scholar, and Semantic Scholar. Pooled prevalence estimates with corresponding 95% confidence intervals (CIs) were calculated using an inverse-variance random-effects model, as substantial heterogeneity was anticipated and confirmed (I^2^ ≥ 50%). Subgroup analyses were performed to report prevalence estimates stratified by refraction method, age group, study setting, and sex.

**Results:**

Twenty-four studies involving 19,331 participants were analysed. Pooled prevalence of total refractive error, myopia, hyperopia and astigmatism were 19.0% (95% CI: 16.0% – 22.1%), 7.1% (95% CI: 5.5%–8.6%), 4.4% (95% CI: 3.4%–5.4%) and 6.3% (95% CI: 4.8% – 7.9%) respectively. These varied according to method of refraction, age group, study setting and sex, with corresponding estimates of 19.0% (95%CI:16.0, 22.1), 20.3% (95%CI:12.0, 28.6), 19.0% (95%CI:16.0, 22.1), and 14.2% (95%CI: 11.4, 16.1) respectively. Subgroup analyses demonstrated significant variation in myopia prevalence, with higher estimates observed in non-cycloplegic studies at 7.9% (95% CI: 6.0–9.8), among adolescents at 22.4% (95% CI: 7.1–37.7), in school-based studies at 8.8% (95% CI: 6.7–10.8), and among females at 9.5% (95% CI: 5.9–13.1).

**Conclusion:**

Prevalence of refractive errors, including myopia, in Ghana remain key public health concern, and vary substantially according to the refraction method employed, the age group studied, and the study setting. This heterogeneity underscores the influence of methodological and population-specific factors and provides an evidence base for informing context-specific and targeted eye-health interventions.

**Supplementary Information:**

The online version contains supplementary material available at https://doi.org/10.1186/s12886-026-05179-9.

## Background

Refractive errors constitute a major cause of global visual impairment, with marked variability observed both between and within countries [1], as well as across demographic groups [1, 2]. They impose substantial personal, social, and economic burdens impact on affected individuals and societies [3]. Pathophysiologically, refractive errors result from a mismatch between the optical power of the refractive components of the eye and axial length, leading to defocused images on the retina [2]. Management strategies primarily include spectacles, contact lenses and refractive surgery, all of which are clinically effective [4]. However, access to these corrective interventions remains limited in many low and middle income settings [3], contributing to a persistently high and, in some regions, increasing prevalence of uncorrected refractive error and its associated burden.

Refractive errors are optical anomalies of the eye characterised by the inability to focus parallel light rays accurately onto the retinal photoreceptor layer. The principal types of refractive error include myopia, hyperopia and astigmatism. Myopia arises when the eye’s refractive power is excessive relative to its axial length, or when the axial length is increased, resulting in image formation anterior to the retinal plane and consequent reduction in distance visual acuity. In contrast, hyperopia occurs when the eye’s refractive power is insufficient or the axial length is reduced, causing images to be focused posterior to the retina and leading to blurred vision, particularly at near distances. Accordingly, individuals with myopia experience greater blur for distant objects than for near objects, whereas individuals with hyperopia typically exhibit the opposite pattern. Astigmatism is caused by unequal refractive power across different meridians of the optical system, leading to the formation of focal lines or planes rather than a single focal point on the retina, leading to the formation of focal lines or planes rather than a single focal point on the retina, and resulting in blurred or distorted vision at both near and far distances. Of these refractive errors, myopia is consistently reported as the most prevalent worldwide [5, 6].

The global prevalence of myopia is increasing at an unprecedented rate, with populations of Asian ancestry disproportionately affected compared with other racial and ethnic groups worldwide [7]. It was estimated that approximately 28% of the global population was myopic in 2010, and the projections indicate that this prevalence will increase to nearly 50% by 2050 [8], positioning myopia as a major and escalating global public health concern [9, 10]. The observed rise in myopia prevalence has been attributed to a complex interplay of genetic susceptibility, racial predisposition, younger age at onset, and, more prominently, modifiable environmental and lifestyle factors, including increased time spent indoors, sustained near work activities, and extensive use of digital devices, particularly among children and adoloscents [11–13]. Notwithstanding these trends, accumulating evidence demonstrates that myopia onset and progression can be effectively mitigated through optical and pharmacological interventions, with even greater impact achieved through the adoption of favourable environmental and behavioural modifications [14–17].

In Africa, the prevalence of myopia remains comparatively low relative to other global regions, particularly East and Southeast Asia [18–21], where prevalence rates range of 80% to 90% have been reported among young urban populations [22]. Current estimates indicate myopia prevalence rates of 1.7% in Ghana and 2.6% in Ethiopia [1, 23]. However, these relatively low figures should not be interpreted as evidence that the continent is insulated from the growing burden of myopia. Exposure to established myopia risk factors is increasing substantially across Africa. By the end of 2020, sub-Saharan Africa had an estimated 495 million mobile phone subscribers, representing approximately 46% of the population, with projections suggesting an increase to 615 million subscribers (50% of population) by 2025 [24]. Increased smartphone use has been identified as a significant risk factor for the onset and progression of myopia [25]. Moreover, rising levels of urbanization and increasing life expectancy, both of which are strongly associated with higher myopia prevalence; are accelerating across the continent [26]. These trends warrant urgent public health attention given the broad visual, educational, and socioeconomic consequences of myopia. If current trajectories persist, the prevalence of myopia in Africa is projected to increase markedly, from approximately 7% in 2010 to 28% by 2050 [7].

Ghana, a sub-Saharan African country in West Africa, is administratively organised into 16 regions, encompassing six metropolitan areas, 77 municipalities and 178 districts. The national population is estimated at 30,832,019, with females constituting 50.7% and males 49.3% of the population. The overall literacy rate is 69.8%, with a higher prevalence among males (74.1%) compared with females (65.6%). Literacy levels are also markedly higher in urban populations than in rural communities. Ghana is undergoing rapid socioeconomic and technological transformation, characterised by increased digitalisation and widespread adoption of smartphones and related visual display technologies. Prolonged exposure to near-work activities and digital screens has been associated with an elevated risk of myopia development [27], underscoring the urgency for early preventive and public-health interventions to mitigate a potential future myopia burden. Furthermore, uncorrected refractive errors remain the leading cause of visual impairment in Ghana, contributing approximately 44.4% of the total visual-impairment burden [28], thereby highlighting a critical and persistent public-health challenge.

The reported prevalence of myopia and other refractive errors in Ghana shows considerable heterogeneity across studies. One population-based study [29] documented a myopia prevalence of approximately 1.7% among school-aged children, while a separate study conducted in a population with comparable demographic characteristics reported nearly double this prevalence [30]. The observed differences between the two studies may be explained, at least in part, by the contrasting study settings. One study was conducted in under-resourced communities, whereas the other was undertaken in private schools. These distinct educational contexts are likely to differ substantially in socioeconomic status, access to educational resources, learning environments, and academic intensity. Such factors may influence visual behaviours, including the amount of near-work and time spent outdoors, which are recognised determinants of refractive error development. Consequently, differences in study setting may have contributed to the variation in the reported prevalence of refractive error between the two studies. In contrast, a substantially higher prevalence of myopia (54%) was observed among self-reporting patients attending eye-care facilities, reflecting differences in study design, sampling methods, and population characteristics [31]. Collectively, these findings suggest that myopia represents an emerging public concern in Ghana, warranting timely and targeted interventions to mitigate associated visual impairment and long-term ocular complications. However, the marked variability in reported prevalence estimates complicates accurate assessment of the national burden of myopia, thereby constraining evidence-based policy formulation and resource allocation. A systematic review is therefore essential to synthesise existing evidence and provide a robust and comprehensive estimate of the prevalence of refractive errors in Ghana. Accordingly, this systematic review aims to determine the prevalence of refractive errors in Ghana, with particular emphasis on myopia.

## Methods

The systematic review was conducted in accordance with the 2020 Preferred Reporting Items for Systematic Reviews and Meta-Analysis (PRISMA) [32] guidelines. The review protocol was registered with the Prospective Register of Systematic Reviews (PROSPERO) under the registration number CRD42022377958.

### Information sources and search strategy

A comprehensive and systematic literature search was independently conducted by two reviewers using relevant Medical Subject Heading (MeSH) and associated sub-headings. The search was performed across five electronic databases: PubMed, ProQuest, Scopus, Google Scholar and Semantic Scholar. To ensure comprehensive coverage, all peer-reviewed publications reporting on refractive errors and/or myopia in Ghana were considered, with no restrictions on publication date or participant age. The search strategy employed appropriate Boolean combinations of the following keywords: “prevalence” OR “magnitude” OR “burden” OR “epidemiology” AND “refractive error” OR “myopia” OR “hyperopia” OR “astigmatism” OR “visual impairment” OR “farsighted” OR “nearsighted” OR “presbyopia” OR “blindness.” The scope of the review was limited to online, full-text, peer-reviewed articles that reported prevalence data on refractive errors in Ghana.

### Eligibility criteria

Studies were eligible for inclusion if they satisfied the following criteria:

i. publication as peer-reviewed journal articles;
ii. investigation of the prevalence of refractive errors and/or myopia within populations in Ghana;
iii. availability in the English language or provision of an English translation;
iv. adoption of a cross-sectional study design;
v. clear description of the sampling strategy employed;
vi. explicit reporting of the refractive error assessment method, including the use of cycloplegic or non-cycloplegic refraction or fogging, and whether measurements were obtained objectively or subjectively; and.
vii. specification of the diagnostic criteria used to define myopia.

Refractive errors were defined as myopia, hyperopia and astigmatism. Studies were excluded if they were clinic-based, commentaries, non-peer-reviewed publications, inaccessible to authors, lacked a clearly defined study population, or determined refractive status solely using pinhole visual acuity. Discrepancies arising during the search process and article eligibility assessment were resolved through consensus by two independent adjudicators.

**Fig. 1:**
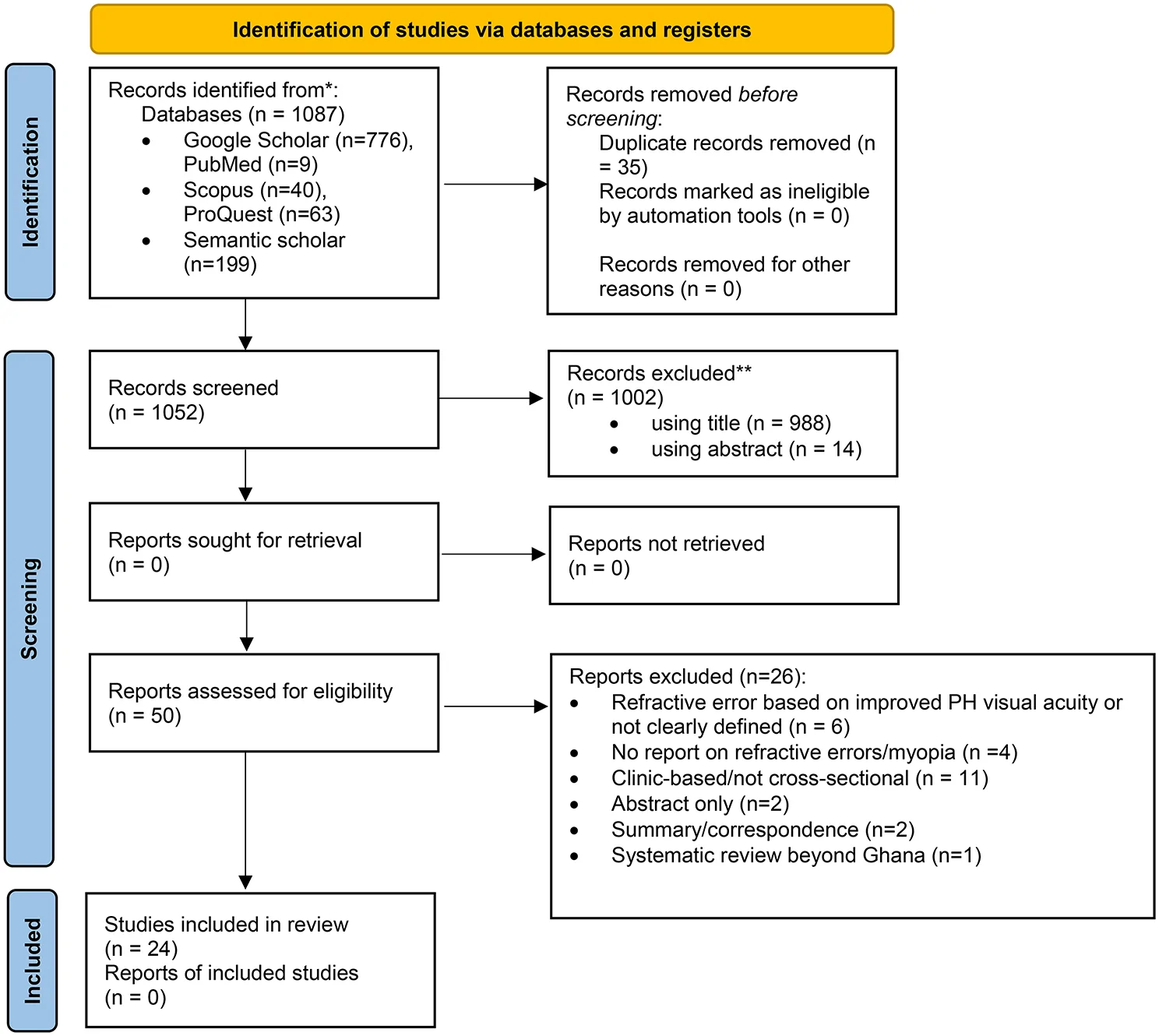
PRISMA 2020 Flow chart of the process of selecting articles for the review [32]

### Selection process, data extraction and data items

The records retrieved from each database were exported to EndNote version 20.2.1 (Build 15749; Clarivate Analytics, USA) for duplicate identification and removal, as well as for screening and study selection. A three-stage screening process was undertaken. In the first stage, titles were screened to exclude articles that were clearly irrelevant to the study topic. In the second stage, abstracts of the remaining records were retrieved, and studies meeting the eligibility criteria were retained. In the final stage, full text articles were assessed in detail, and studies that satisfied the predefined eligibility criteria were included in the review. Data extraction and methodological appraisal were conducted independently by two reviewers. Any discrepancies were resolved through discussion and, where necessary, consultation with an independent adjudicator to achieve consensus. The final set of included studies was exported to Microsoft Excel for data extraction. The extracted variables included author(s), year of publication, study setting, study design, study population, sample size, age characteristics, sex distribution, criteria used to define refractive errors, and the reported prevalence of refractive errors, including myopia, hyperopia, astigmatism.

### Risk of bias assessment

The methodological quality of the included studies was assessed using the Downs and Black checklist for quality assessment, adapted from a previously published study [23]. Each eligible study was independently evaluated using a 10-item scoring scale, with studies achieving a score of ≥ 6 deemed to be of sufficient quality for inclusion. Quality appraisals were conducted independently by two reviewers, and any disagreements discussed until consensus was reached.

### Reliability

A second reviewer (CNY), blinded to the primary reviewer (SK), independently evaluated the relevance of all identified articles and cross-checked the extracted data for accuracy. Study eligibility was assessed at each stage of the review process. Where there were discrepancies between reviewers, resolution was reached through an independent third reviewer (KEA), to determine the final inclusion of studies.

### Effect measures, categorisation of the study outcomes and synthesis

The primary outcome of this study was the pooled prevalence of myopia and other refractive errors, estimated using random-effects meta-analysis models. Refractive errors were defined as myopia (spherical equivalent < − 0.50 dioptres), hyperopia (spherical equivalent > + 0.50 dioptres), and astigmatism (cylindrical error < − 0.50 dioptres). Overall refractive error prevalence was calculated as the combined prevalence of myopia, hyperopia, and astigmatism.

Subgroup analyses were conducted to explore variations in prevalence according to age, sex, method of refraction (classified as cycloplegic, non-cycloplegic, or fogging), and study setting (school-based or community-based). Owing to substantial heterogeneity in age categorisation across included studies, prevalence estimates were stratified into mid-childhood (5–10 years) and adolescence (11–20 years), in accordance with World Health Organization and UNICEF age classifications [33, 34].

### Statistical analysis

The extracted data were captured in Microsoft Excel and subsequently exported to Stata version 15.0 (StataCorp LLC, College Station, TX, USA) for statistical analysis. An initial descriptive summary was performed to characterise the distribution of studies according to myopia and other refractive errors. Meta-analyses of prevalence were conducted using the *metaprop* command in Stata to estimate study-specific and pooled prevalence estimates with corresponding 95% confidence intervals (CIs). Subgroup analyses were undertaken for myopia and other refractive errors. Between-study heterogeneity was assessed using the I^2^ statistic, which quantifies the proportion of total variability attributable to heterogeneity rather than chance. Heterogeneity was considered statistically meaningful when the I² value was ≥ 50%, indicating substantial inconsistency across studies.

Differences in subgroup prevalence across categories were evaluated using two-sample tests of proportions, implemented in Stata with the “prtesti” command. These analyses compared category-specific point estimates and produced absolute differences accompanied by 95% CI. Statistical significance for within-subgroup comparisons was determined using a two-sided p-value threshold of < 0.001.

## Results

A comprehensive literature search yielded 1087 potentially relevant records. As depicted in the study selection flow diagram (Fig. 1), 50 full-texts articles were assessed for eligibility, of which 24 satisfied the predefined inclusion criteria.

### Study characteristics

The included studies collectively comprised 19,331 participants, with individual sample sizes ranging from 85 to 5603, and were published between 2010 and 2022. All studies utilised variations of a cross-sectional study design. Specifically, nine were descriptive, three analytical, three population or community-based, two prospective, one observational, and six were reported simply as cross-sectional without further methodological specification. With regard to refractive assessment, only two studies employed cycloplegic refraction, while an additional two studies used fogging techniques, the remaining studies did not apply either method. Findings relating to refractive error subtypes are presented without emphasis on any specific category, in order to minimise the risk of selective reporting. A comprehensive summary of the characteristics of the included studies, together with their quality assessment scores, is provided in Table 1.

**Table 1:** Characteristics of papers that reported the prevalence of refractive errors and or including myopia among study populations

Author	Year	Age group	Mean age ± sd	Sample size	Study setting	Type of refraction	Prev. of refractive error	Prev. of myopia	Prev. of hyperopia	Prev. of astigmatism	Quality Assessment Score
Asare and Morjaria	2021	12–15	14.1 ± 0.9	1705	school-based	non-cyclo	1.8%	0.8%	0.4%	0.6%	10
Kumah et al.,	2020	5–18	13.0 ± 2.9	400	school-based	fogging	25.3%	2.5%	18.5%	4.3%	6
Abu et al.	2015	9–22	14.8 ± 0.5	1029	school-based	non-cyclo	3.6%	1.5%	1.3%	0.9%	10
Nakua et al.	2015	12–17	-	504	school-based	non-cyclo	7.5%	2.2%	2.4%	3.0%	10
Ovenseri-Ogbomo et al.	2013	9–27	15.9 ± 4.0	235	school-based	non-cyclo	31.9%	5.5%	2.1%	24.3%	10
Ovenseri-Ogbomo and Assien,	2010	11–18	14.5 ± 1.5	595	school-based	non-cyclo	13.3%	1.7%	5.0%	6.6%	9
Ovenseri-Ogbomo and Omuemu,	2010	5–19	10.5 ± 3.4	957	school-based	cyclo	25.6%	6.9%	4.6%	14.1%	9
Kumah et al.	2016	14–20	16.2 ± 2.6	662	school-based	non-cyclo	-	25.1%	-	-	10
Kumah et al.	2013	12–15	-	2435	school-based	cyclo	3.3%	3.4%	0.3%	13.7%	10
Nartey et al.	2016	6–16	10.6	811	school-based	non-cyclo	-	4.8%	1.6%	0.4%	10
Boitey and Amankwah	2017	12–25	17.5	202	school-based	non-cyclo	-	27.2%	-	-	9
Afari	2014	18–99	46.3 ± 0.5	1300	community-based	non-cyclo	7.6%	4.4%	3.2%	-	10
Abdul-Kabir et al.	2022	17–27	18.6 ± 1.4	85	school-based	non-cyclo	68.2%	40.0%	28.2%	-	9
Darko-Takyi et al.	2018	12–17	14.1 ± 1.5	202	school-based	non-cyclo	18.8%	4.5%	5.0%	9.4%	10
Abu et al.	2016	15–70	35.5 ± 11.3	433	community-based	non-cyclo	6.7%	0.5%	1.2%	5.1%	10
Boadi-Kusi et al.	2016	20–75	39.2 ± 11.0	520	community-based	non-cyclo	20.4%	1.9%	12.7%	5.8%	10
Abdul-Kabir et al.	2014	13–17	-	204	school-based	non-cyclo	14.2%	-	-	-	9
Boadi-Kusi	2013	19–70	52.7 ± 11.7	185	community-based	non-cyclo	28.6%	-	-	-	10
Budenz et al.,	2012	40-≥81	52.7 ± 10.9	5603	community-based	non-cyclo	10.9%	-	-	-	10
Tanle et al.,	2011	< 25-≥55		100	community-based	fogging	32.5%	7.0%	9.0%	16.5%	9
Ovenseri-Ogomo and Adofo	2011	18–68	39.2 ± 11.8	206	community-based	non-cyclo	32.0%	5.8%	10.7%		9
Darko-Takyi et al.	2016	19–27	22.6 ± 1.5	105	school-based	non-cyclo	59.0%	17.1%	19.0%	22.9%	10
Abdul-Kabir et al.	2016	10–15	-	208	school-based	non-cyclo	30.3%	22.6%	2.4%	5.3%	10
Opoku Yamoah et al.	2021	6–16	11.1 ± 3.1	645	school-based	non-cyclo	9.8%	6.8%	2.0%	0.9%	10

### Prevalence of refractive error types

**Fig. 2:**
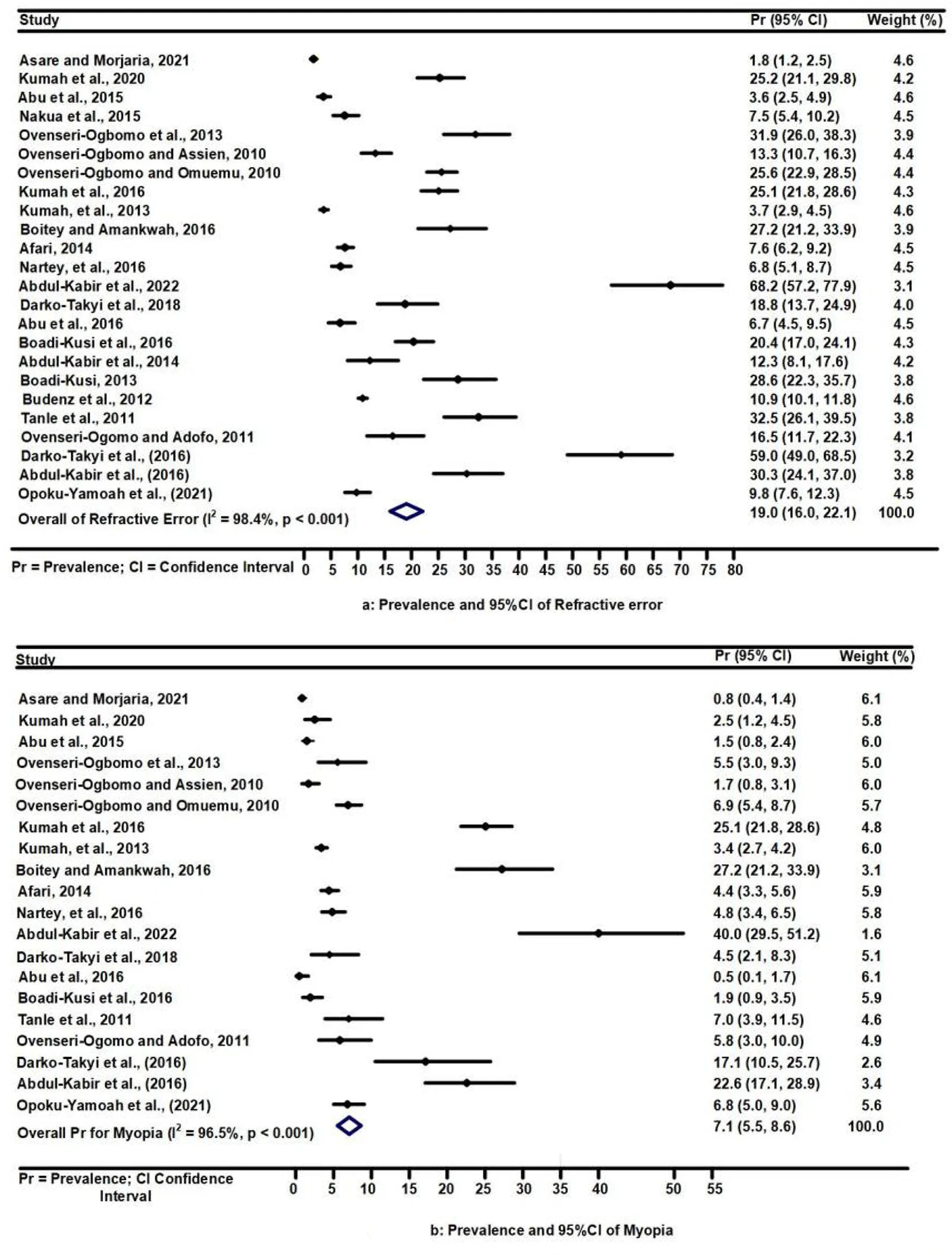

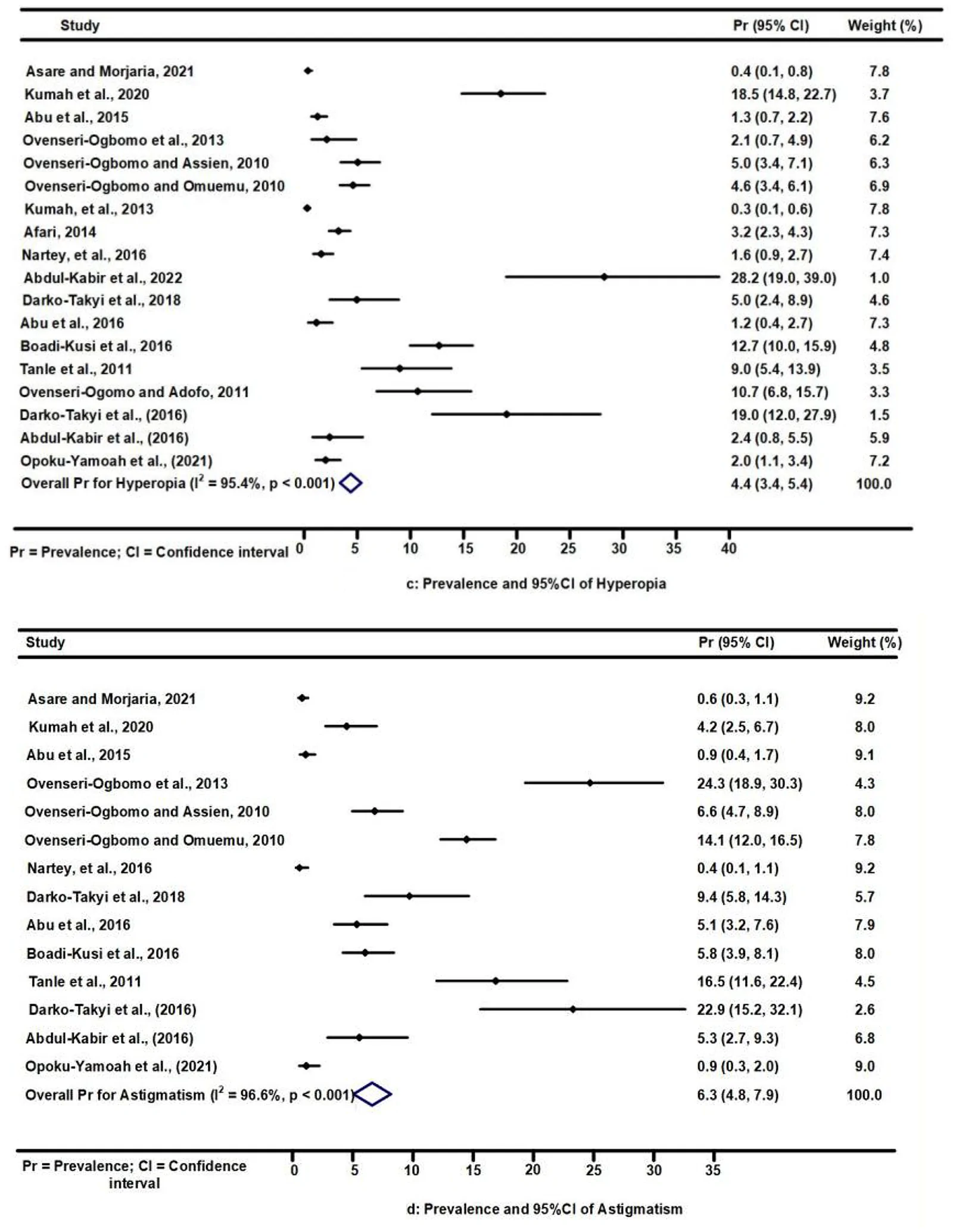
Pooled prevalence and 95% confidence intervals of refractive error types

Twenty-four studies contributed to the pooled prevalence of total refractive errors, which was 19.0% (95% CI: 16.0% – 22.1%), and ranged from 1.8% to 68.2%, with considerable heterogeneity across studies (I² = 98.4%, *p* < 0.001) (Fig. 2a). Across 20 studies, the pooled prevalence of myopia was 7.1% (95% CI: 5.5%–8.6%). Reported prevalence estimates varied from 0.5% to 40.0%, and there was substantial between-study heterogeneity (I² = 96.5%, *p* < 0.001) (Fig. 2b).

The pooled prevalence of hyperopia, based on 18 studies, was 4.4% (95% CI: 3.4%–5.4%). Individual study prevalence estimates ranged from 0.4% to 28.3%, and there was significant between-study heterogeneity (I² = 95.4%, *p* < 0.001) (Fig. 2c).

Pooling data from 14 studies yielded a pooled prevalence of astigmatism of 6.3% (95% CI: 4.8% – 7.9%). Individual study prevalence estimates ranged from 0.4% to 24.3%, with considerable between-study heterogeneity (I² = 96.6%, *p* < 0.001) (Fig. 2d).

### Refractive error and method of refraction

**Fig. 3:**
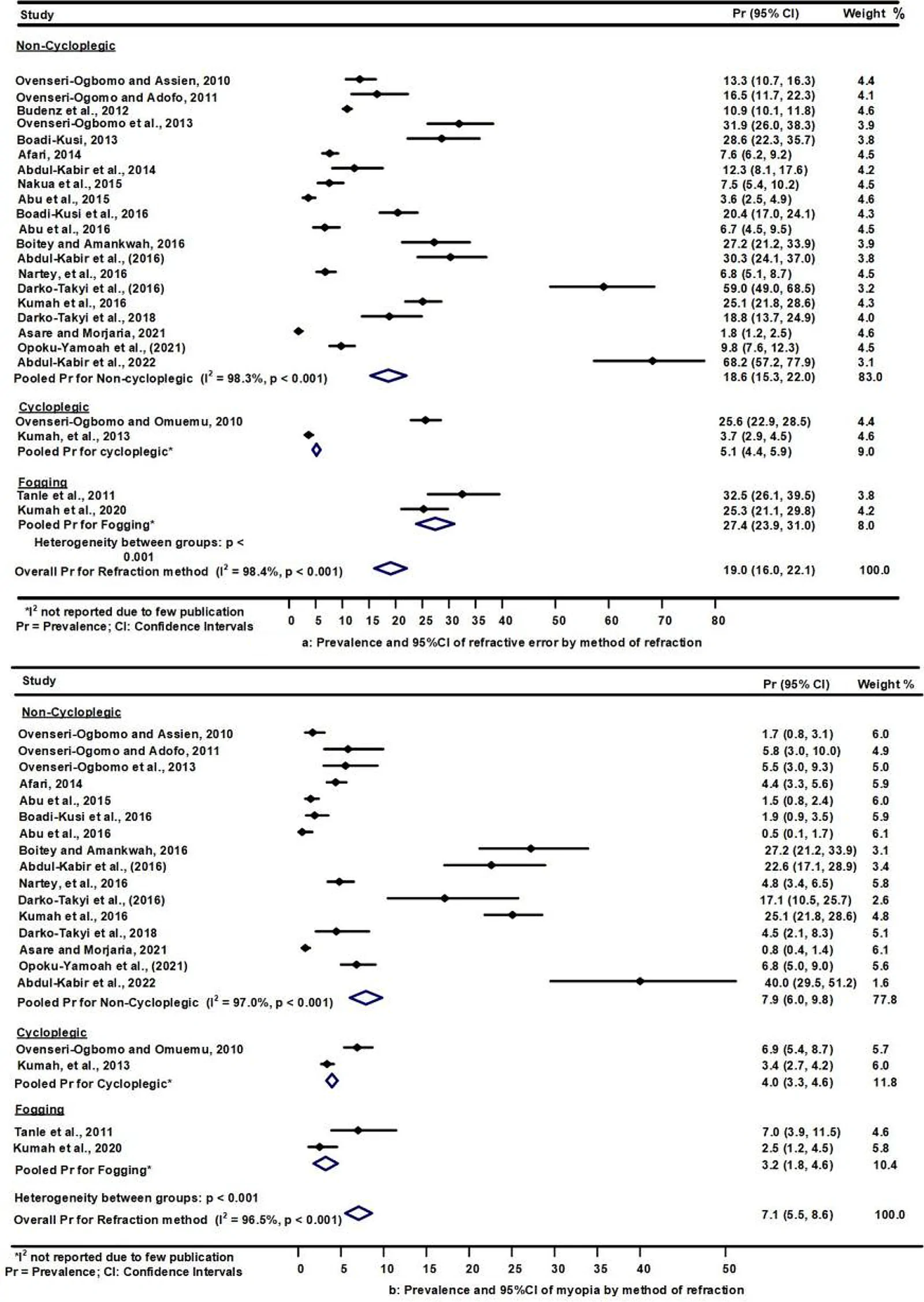

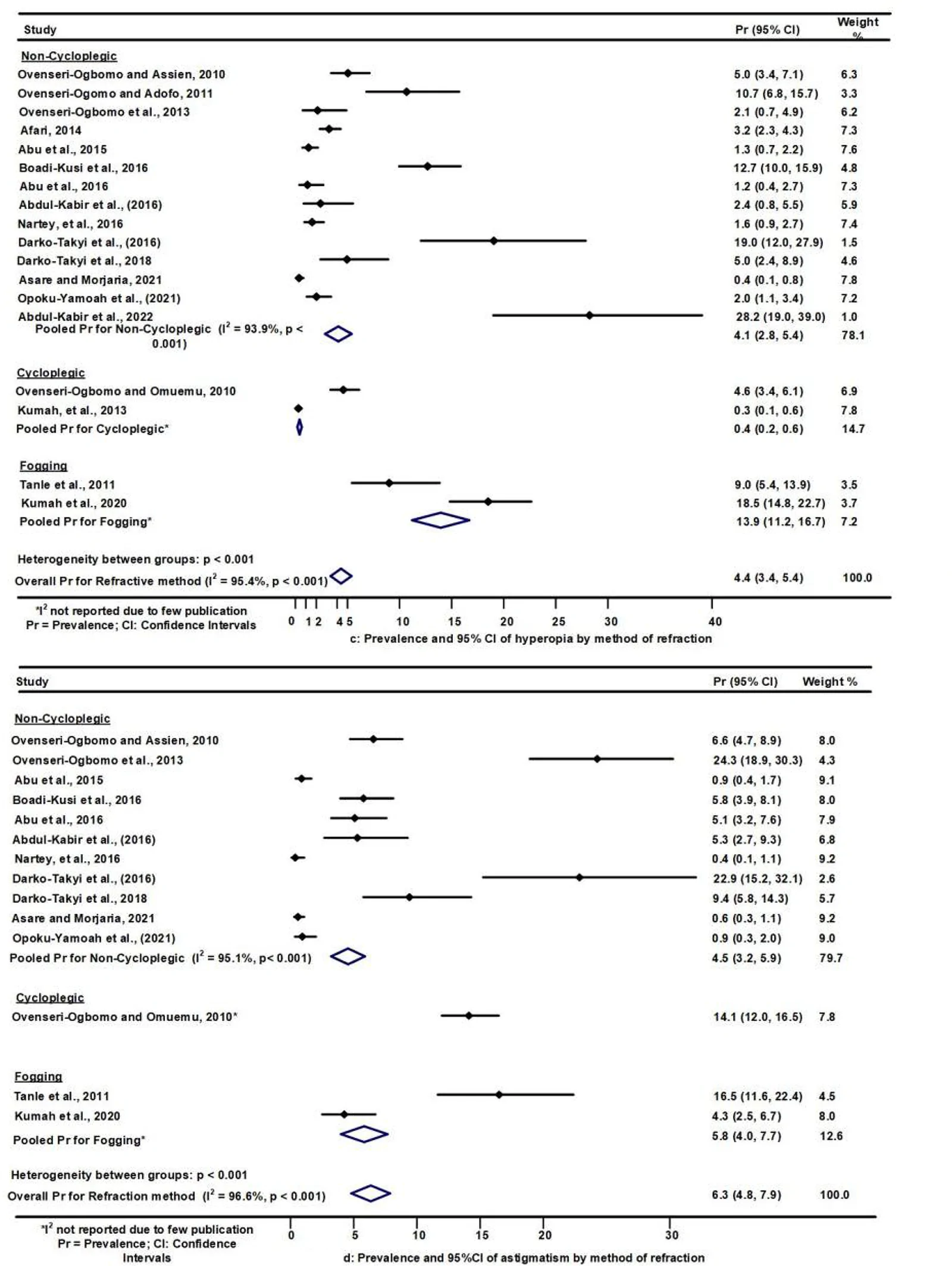
Refractive errors and method of study

The prevalence among studies employing non-cycloplegic refraction was 18.6% (95%CI:15.3, 22.0), whereas studies using cycloplegic refraction reported a substantially lower pooled prevalence of 5.1% (95%CI:4.4, 5.9). Studies that conducted refraction under fogging conditions demonstrated a prevalence of 27.4% (95%CI; 23.9, 31.0) [Figure 3a]. The prevalence difference between non-cycloplegic and cycloplegic refraction was 13.5% (95% CI: 12.5–14.5, *p* < 0.001). The difference between cycloplegic refraction and fogging was 22.3% (95% CI: 18.5–25.9, *p* < 0.001), while the difference between non-cycloplegic refraction and fogging was 8.8% (95% CI: 5.2–12.4, *p* < 0.001). Studies that did not employ cycloplegic refraction demonstrated significant heterogeneity (I^2^ = 98.3%, *p* < 0.001) with prevalence estimates ranging from a high of 68.2% (95%CI:57.2, 77.9) to a low of 1.8% (1.2, 2.5) (Fig. 3a).

Subgroup analyses demonstrated significant myopia prevalence variation according to refractive assessment method. Studies employing non-cycloplegic refraction reported a higher prevalence of 7.9% (95% CI: 6.0–9.8) compared with those using cycloplegic refraction, which yielded a prevalence of 4.0% (95% CI: 3.3–4.6). Studies utilising the fogging technique reported the lowest prevalence at 3.2% (95% CI: 1.8–4.6) (Fig. 3b). Pairwise comparisons indicated a statistically significant difference of 3.9% (95% CI: 3.0–4.8; *p* < 0.001) between non-cycloplegic and cycloplegic refraction. In contrast, the difference between cycloplegic refraction and fogging was not statistically significant (0.8%, 95% CI: −0.1–2.4; *p* = 0.350). The difference between non-cycloplegic refraction and fogging was statistically significant at 4.7% (95% CI: 3.2–6.2; *p* < 0.001).

Regarding hyperopia, studies employing non-cycloplegic refraction reported a prevalence of 4.1% (95% CI: 2.8–5.4), whereas those using cycloplegic refraction reported a markedly lower prevalence of 0.4% (95% CI: 0.2–0.6). In contrast, studies utilising fogging techniques reported a substantially higher prevalence of 13.9% (95% CI:11.2–16.7) (Fig. 3c). Subgroup analysis demonstrated statistically significant differences in hyperopia prevalence across refraction methods. The prevalence differed by 3.7% (95% CI: 3.2–4.2; *p* < 0.001) between non-cycloplegic and cycloplegic refraction. The difference between cycloplegic refraction and fogging was 13.5% (95% CI: 10.7–16.3; *p* < 0.001), while the difference between non-cycloplegic refraction and fogging was 9.8% (95% CI: 6.9–12.6; *p* < 0.001).

Stratified analyses by refraction method showed that studies employing non-cycloplegic refraction reported an astigmatism prevalence of 4.5% (95% CI: 3.2–5.9), whereas those using cycloplegic refraction reported a markedly higher prevalence of 14.1% (95% CI: 12.0–16.5). Studies adopting the fogging technique yielded an intermediate prevalence of 5.8% (95% CI: 4.0–7.9). Subgroup comparisons demonstrated a statistically significant absolute difference in astigmatism prevalence of 9.6% (95% CI: 7.3–11.9; *p* < 0.001) between non-cycloplegic and cycloplegic refraction. Similarly, the difference between cycloplegic refraction and the fogging technique was significant at 8.3% (95% CI: 5.4–11.3; *p* < 0.001). In contrast, the difference between non-cycloplegic refraction and the fogging technique was small and not statistically significant (1.3%; 95% CI: −0.1 to 3.2; *p* = 0.146).

### Refractive errors and age group

**Fig. 4:**
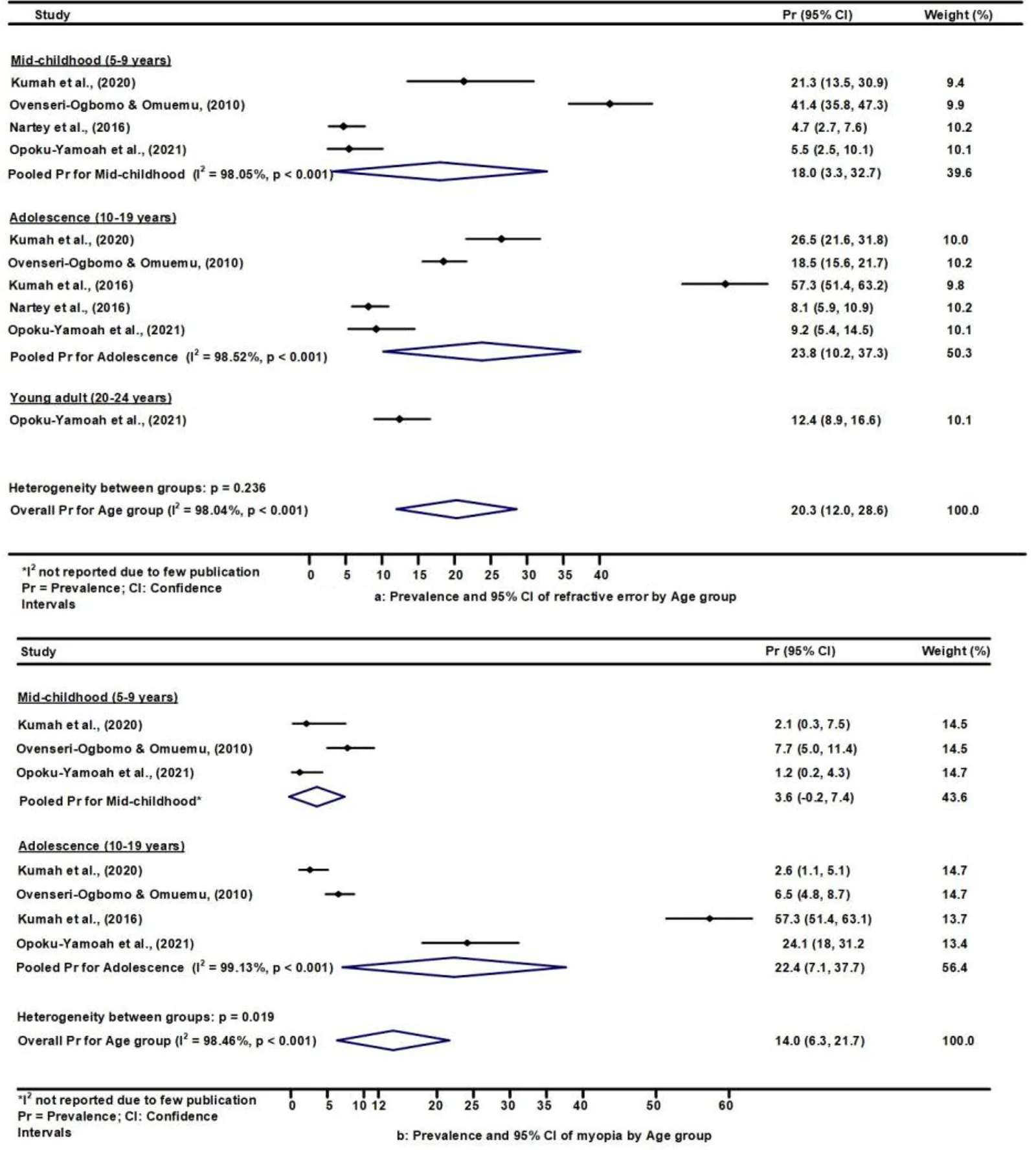

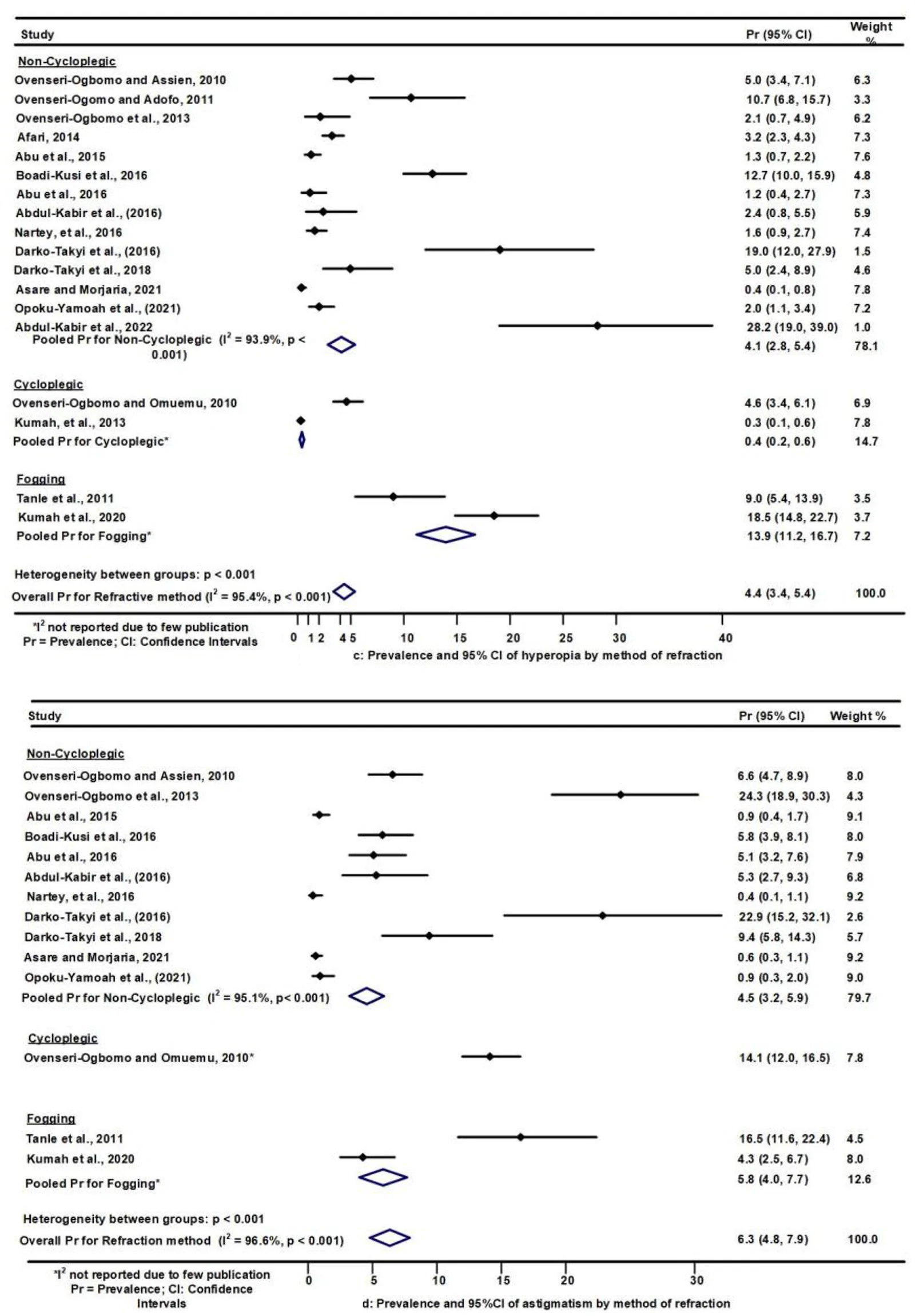
Refractive errors and age group

The overall prevalence of total refractive error across age groups was 20.3% (95% CI: 12.0–28.6). Age-stratified analyses demonstrated a prevalence of 18.0% (95% CI: 3.3–32.7) in mid-childhood (5–9 years), 23.8% (95% CI: 10.2–37.3) in adolescence (10–19 years), and 12.4% (95% CI: 8.9–16.6) in young adulthood (20–24 years). The variation in refractive error prevalence across age groups was statistically significant, with an absolute difference of 5.8% (95% CI: 2.9–8.7; *p* = 0.002). However, no significant heterogeneity was observed between age groups (*p* = 0.236), indicating consistency in effect estimates (Fig. 4a).

The prevalence of myopia based on age was estimated at 14.0% (95% CI: 6.3–21.7), with mid-childhood prevalence of 3.6% (95% CI: −0.2–7.4) and22.4% (95% CI: 7.1–37.7) for adolescence. The prevalence of myopia differed significantly across age groups, with an absolute difference of 18.8% (95% CI: 16.7%–20.9%; *p* < 0.001). In addition, significant between-study heterogeneity (*p* = 0.019) and substantial variability were observed (Fig. 4b).

The prevalence of hyperopia across all studies, based on age was 9.0% (95% CI: 4.0–14.1) (Fig. 4c). Hyperopia was more prevalent in mid-childhood, 10.4% (95% CI: 2.9–17.8) than in adolescence, 7.8% (95% CI: −0.2–15.8). The prevalence of hyperopia demonstrated a modest but statistically significant difference between mid-childhood and adolescence was 2.6% (95% CI: 1.0%–5.3%; *p* = 0.039). There was no evidence of statistically significant heterogeneity between age groups (*p* = 0.641).

A comparable non-significant pattern was observed for astigmatism (*p* = 0.872), with an overall prevalence of 6.7% (95% CI: 2.2–11.2) (Fig. 4d). The prevalence in mid-childhood was 7.5% (95% CI: −0.4 to 15.3), compared with 6.2% in adolescence (95% CI: 1.7–10.7) (Fig. 4d). There was no statistically significant difference in astigmatism prevalence between age groups (difference: 1.3%; 95% CI: −1.0 to 3.6; *p* = 0.248).

Funnel plots were generated and Begg’s rank correlation test was applied to assess potential publication bias for refractive error, myopia, hyperopia, and astigmatism across age-group-specific categories. The analyses demonstrated no publication bias, as shown in Supplementary Figure 1.

### Refractive error prevalence and study site

**Fig. 5:**
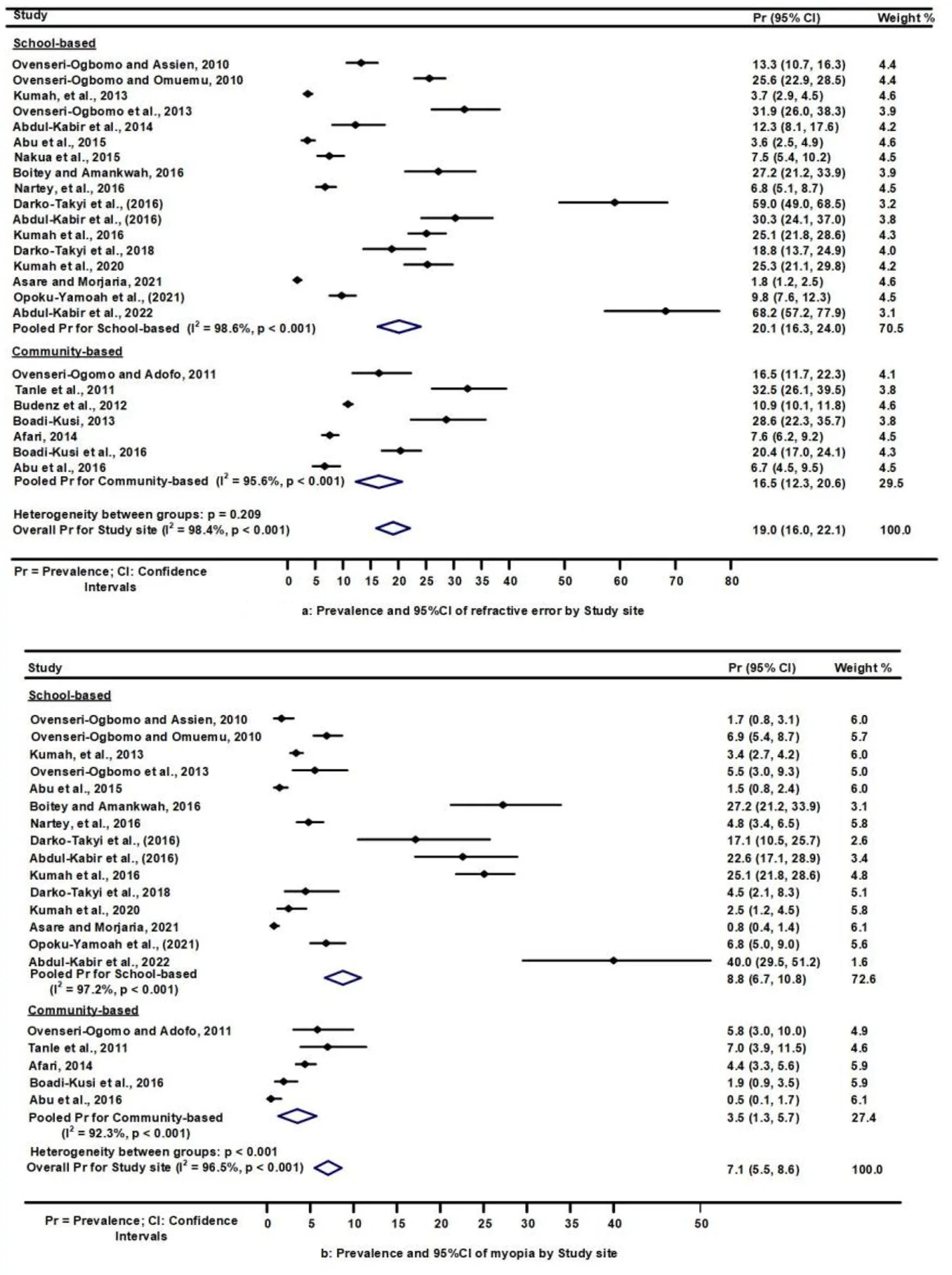

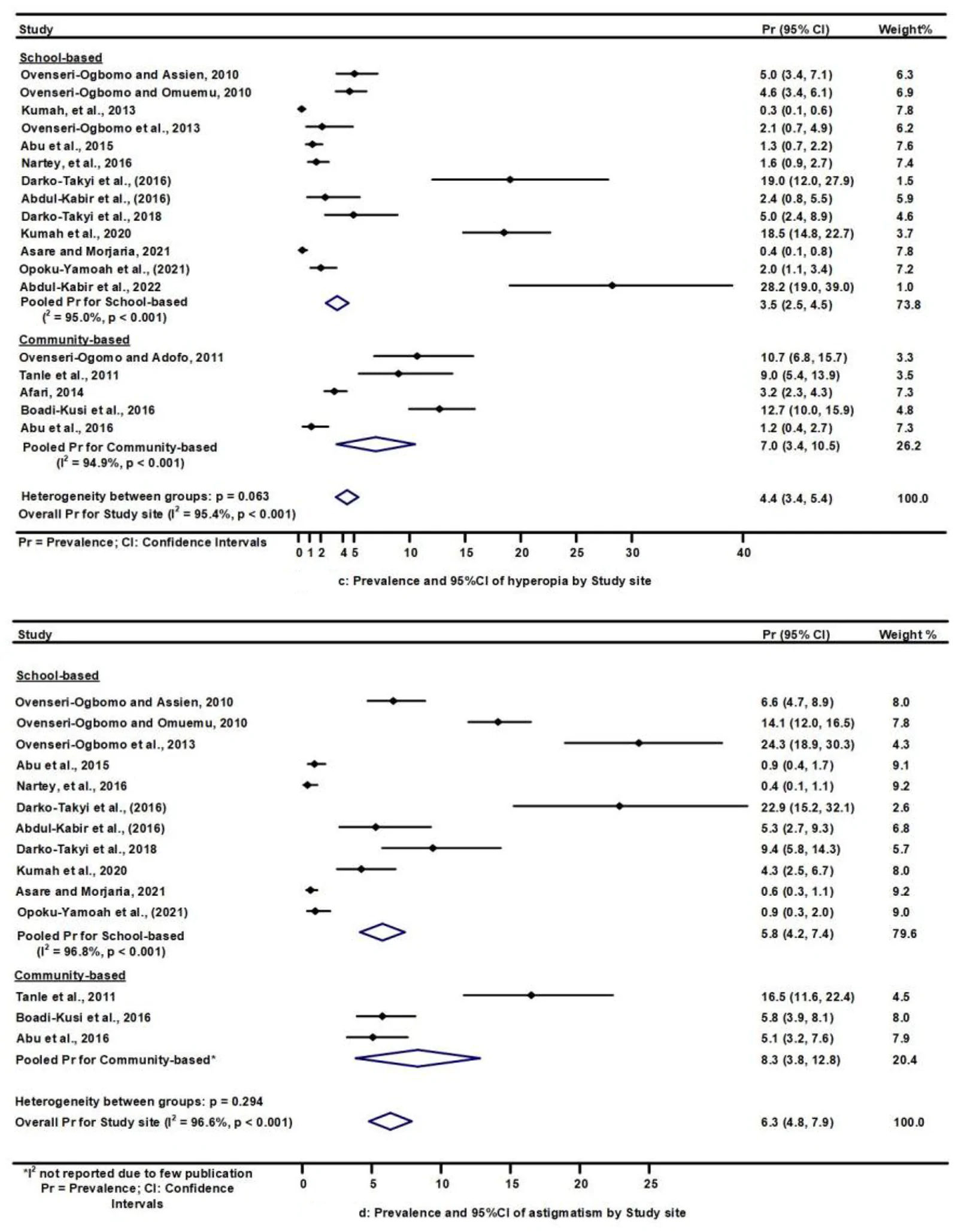
Refractive errors and study site

Subgroup analysis by study setting showed a prevalence of 20.1% (95% CI: 16.3–24.0) in school-based studies and 16.5% (95% CI: 12.3–20.6) in community-based studies (Fig. 5b). The prevalence difference between School-based and community-based studies was 3.4% for refractive error (95% CI: 2.3%–4.5%, *p* < 0.001).

Regarding myopia, a significantly higher prevalence was observed in school-based studies at 8.8% (95% CI: 6.7–10.8) compared with community-based studies, which reported a prevalence of 3.5% (95% CI: 1.3–5.7) (Fig. 5b). The myopia prevalence difference between School-based and community-based studies was 5.3% (95% CI: 4.4%–6.1%; *p* < 0.001).

This difference was accompanied by substantial heterogeneity across studies (*p* < 0.001) Subgroup analysis further demonstrated a statistically significant absolute difference in myopia prevalence of between school-based and community-based study settings.

School-based studies yielded hyperopia prevalence of 3.5% (95% CI: 2.5–4.5), whereas community-based studies reported a higher prevalence of 7.0% (95% CI: 3.4–10.5). No statistically significant heterogeneity was observed between these study settings (*p* = 0.063; Fig. 5c). However, a statistically significant absolute difference of 3.5% (95% CI: 2.5–4.5; *p* < 0.001) in hyperopia prevalence between school-based and community-based studies.

School-based studies reported an astigmatism prevalence of 5.8% (95% CI: 4.2–7.4), whereas community-based studies reported a higher prevalence of 8.3% (95% CI: 3.8–12.8). The absolute difference in astigmatism prevalence between school-based and community-based studies was 2.5% (95% CI: 0.8–4.2), which was statistically significant (*p* = 0.001), with no evidence of heterogeneity (*p* = 0.294) (Fig. 5d).

Publication bias was assessed using funnel plots and Begg’s test for refractive error overall, myopia, hyperopia, and astigmatism stratified by study site. These analyses demonstrated no evidence of publication bias (Supplementary Figure 2).

### Refractive error prevalence and gender

**Fig. 6:**
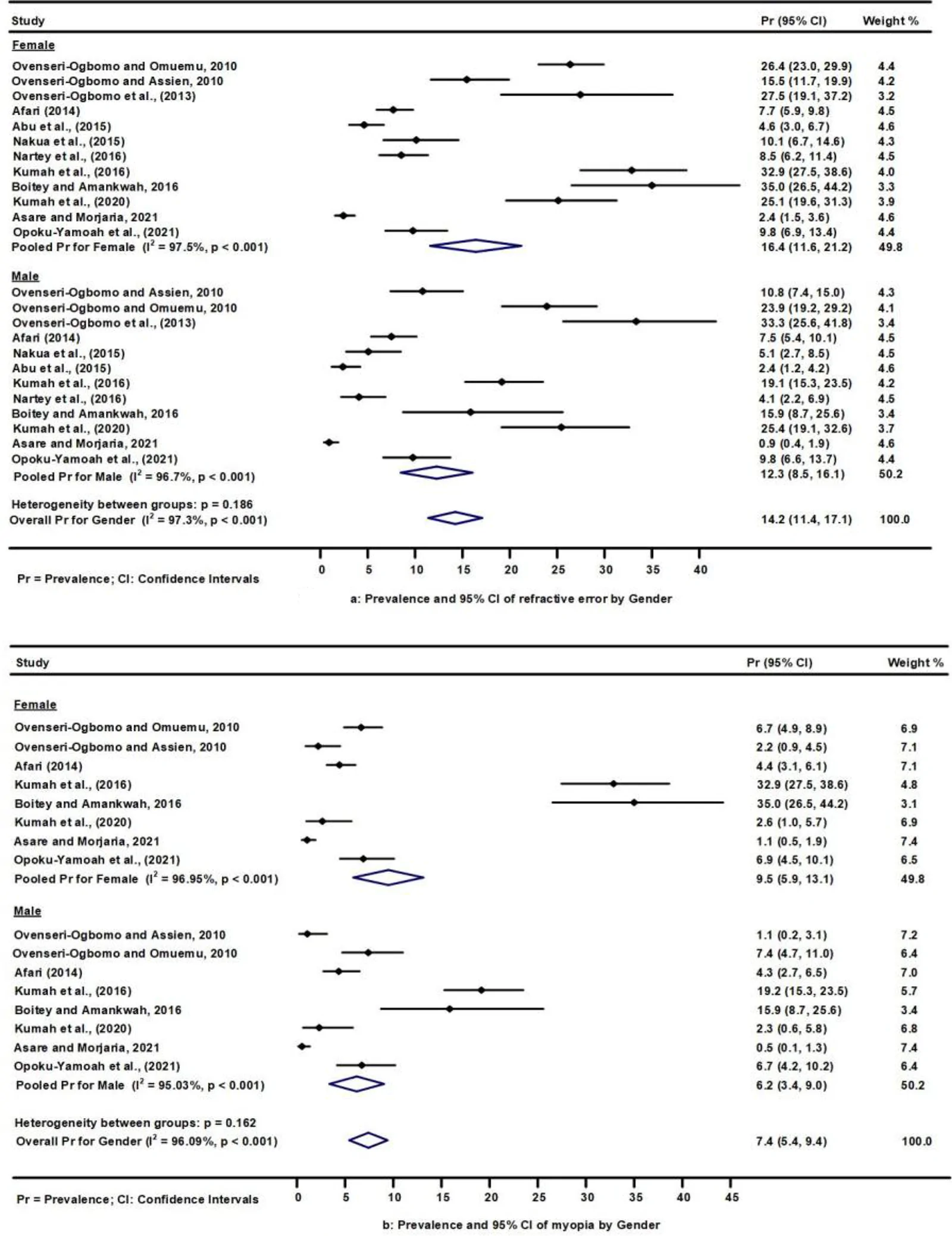

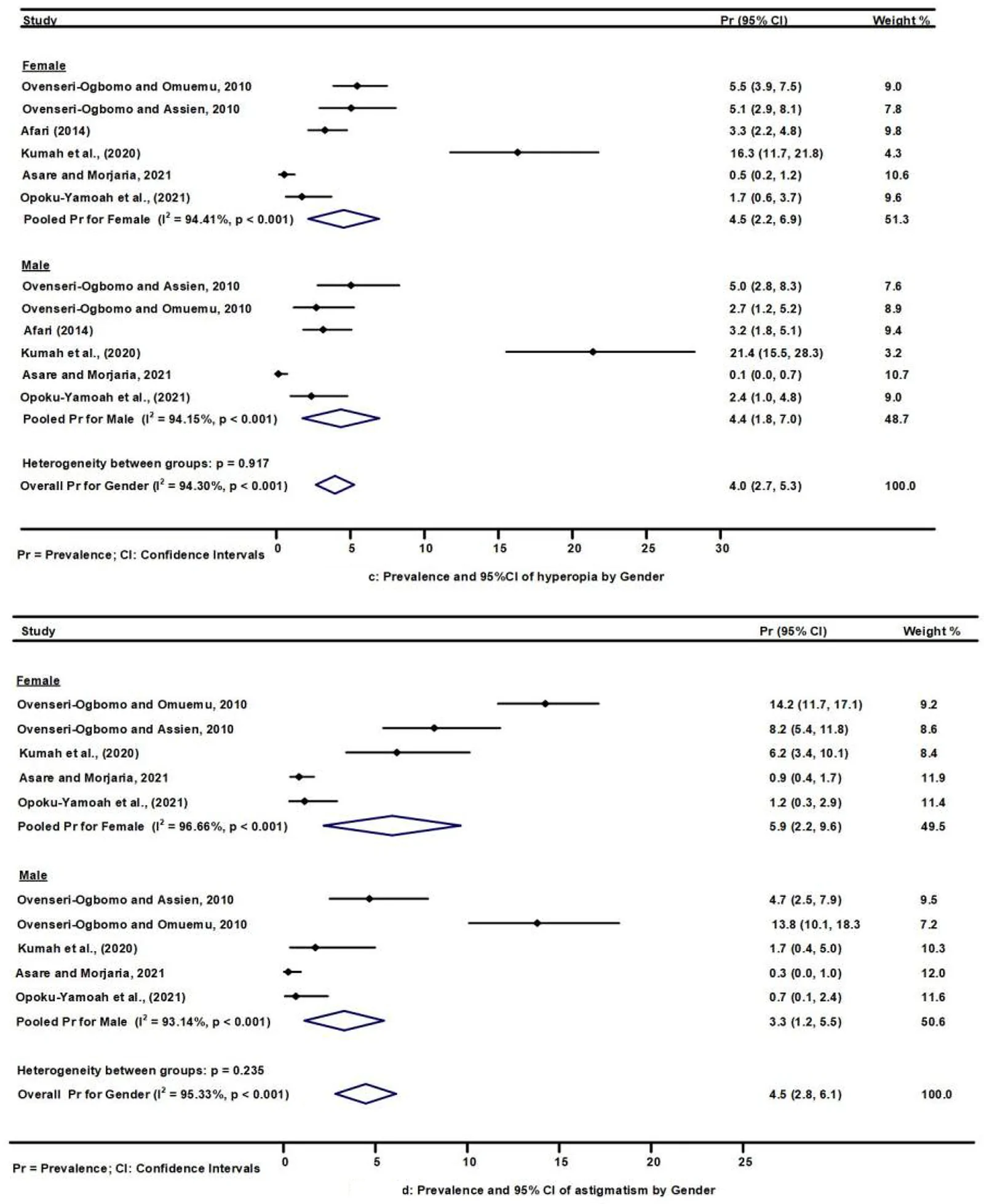
Refractive errors and gender

The overall prevalence of total refractive error based on gender was 14.2% (95% CI: 11.4–17.1). Gender stratified prevalence was 16.4% among females (95% CI: 11.6–21.2) and 12.3% among males (95% CI: 8.5–16.1). The comparison between genders showed no statistically significant heterogeneity (*p* = 0.186) (Fig. 6a). The prevalence difference between males and females was 4.1% for refractive error 4.1% (95% CI: 2.6%–5.5%; *p* < 0.001).

The prevalence of myopia based on gender was 7.4% (95% CI: 5.4–9.4). Prevalence for females was 9.5% (95% CI: 5.9–13.1) and males 6.2% (95% CI: 3.4–9.0) (Fig. 6b). The prevalence difference between males and females was 3.3% for myopia (95% CI: 2.0%–4.6%; *p* < 0.001).

The prevalence of hyperopia based on gender was 4.0% (95% CI: 2.7–5.3) (Fig. 6c). Among females, the prevalence was 4.5% (95% CI: 2.2–6.9) and 4.4% (95% CI: 1.8–7.0) among males, with no statistically significant difference between sexes (*p* = 0.917). The prevalence difference between males and females was 0.1% (95% CI: −0.9 to 1.2, *p* = 0.858) which indicates that there was no statistically significant difference in the prevalence of hyperopia between males and females. ().

The prevalence of astigmatism by gender was 4.5% (95% CI: 2.8–6.1). There was no statistically significant difference in prevalence between females 5.9% (95% CI: 2.2–9.6) and males 3.3% (95% CI: 1.2–5.5; *p* = 0.516) (Fig. 6d). The prevalence difference between males and females was 2.6% (95% CI: 1.4%–3.8%; *p* = 0.001).

Funnel plots were constructed, and Begg’s rank correlation test was applied to assess potential publication bias in the estimates of refractive error, myopia, hyperopia, and astigmatism stratified by gender. The analyses revealed no evidence of publication bias for any of the outcomes assessed (Supplementary Figures S1–S4).

### Meta-regression of log proportion of refractive error, myopia, hyperopia, and astigmatism, and the year of publication

A meta-regression analysis examining total refractive error, myopia, hyperopia, and astigmatism as functions of year of publication demonstrated a negative association between publication year and each refractive outcome. Specifically, for each one-unit increase in total refractive error, myopia, hyperopia or astigmatism, there was a corresponding decrease with advancing publication year; however, these associations were not statistically significant (*P* > 0.05). Detailed results are presented in Supplementary Tables S5–S8.

The year of publication was not significantly associated with the proportion of total refractive error. Although the fitted regression line indicated a marginal upward trend, this association was not statistically significant (*p* = 0.828; Supplementary Figure S5).

The proportion of myopia demonstrated a weak negative correlation with publication year; however, this association was not statistically significant (*p* = 0.851) (Supplementary Figure S6). Similarly, the proportion of hyperopia showed a weak negative correlation with publication year, with no statistically significant difference observed (*p* = 0.402) (Supplementary Figure S7). In contrast, the proportion of astigmatism exhibited a moderate positive correlation with publication year, although this trend did not reach statistical significance (*p* = 0.071) (Supplementary Figure S8).

## Discussion

This systematic review and meta-analysis sought to quantify the prevalence of refractive errors in Ghana, with specific emphasis on myopia. To the best of our knowledge, this represents the first comprehensive synthesis of population-based evidence on refractive error prevalence in the country, drawing on data that are not limited to hospital- or facility-based settings. The included studies demonstrated considerable heterogeneity with respect to refraction techniques, age distributions, study locations, and sex. Consequently, in addition to pooled prevalence estimates determined from all studies, specific estimates were derived through subgroup analyses stratified by these demographic and methodological characteristics. Although the review synthesised evidence across all categories of refractive error, the discussion places particular emphasis on myopia, given its documented and growing public health significance.

The findings show that refractive errors remain a substantial public health burden in Ghana, with an overall pooled prevalence of 19.0% and myopia (7.1%) exceeding astigmatism (6.3%) and hyperopia (4.4%). Overall, females had a higher pooled prevalence of total refractive error, myopia, hyperopia, and astigmatism than males. Hyperopia and astigmatism were more prevalent during mid-childhood, whereas total refractive error and myopia were more prevalent during adolescence. In addition, school-based studies reported higher prevalences of hyperopia and astigmatism, while community-based studies reported higher prevalences of total refractive error and myopia. Total refractive error and myopia prevalence estimates from studies employing cycloplegic techniques were lower compared to this employing fogging and non-cycloplegic methods. These findings highlight important demographic and methodological variations in the epidemiology of refractive errors and may help inform targeted vision screening, prevention, and eye care strategies for children and adolescents in Ghana.

The overall prevalence estimate for total refractive error was higher than several child-focused African and country-specific syntheses, among Ethiopian students and Nigerian children, suggesting a broader age mix, different case definitions, or setting-specific risk profiles in the present review [35, 36]. It is also higher than the pooled prevalence reported for Nepalese children, where myopia was likewise the dominant subtype, but remains close to reported estimate for European children, indicating that the present findings are in keeping with contemporary international data, particularly in populations undergoing epidemiologic transition [37, 38]. The finding that myopia was the leading subtype is consistent with African school-based meta-analyses as well as with global projections that childhood myopia now affects about one-third of children and adolescents and continues to rise over time [23, 36, 39]. Differences between reviews likely reflect methodological and population heterogeneity, especially age structure, urbanicity, ethnicity, diagnostic thresholds, and refraction technique, since non-cycloplegic methods consistently overestimate myopia and increase variability relative to cycloplegic refraction in children [23, 38, 40]. The substantial between-study heterogeneity in the present meta-analysis therefore, likely reflects real epidemiologic diversity rather than random error alone, reinforcing that refractive error burden is not uniform across settings [23, 41, 42]. Clinically and from a policy perspective, these findings support routine school- and community-based vision screening, timely spectacle provision, and targeted prevention strategies that increase outdoor time and strengthen access to refractive services, particularly in underserved settings where spectacle coverage remains poor and uncorrected refractive error continues to drive avoidable visual impairment. Future prevalence studies should adopt standardized case definitions and cycloplegic protocols to improve cross-study comparability and produce estimates that are more useful for service planning and public health surveillance.

The prevalence of total refractive error varied significantly according to the refraction method employed. Studies utilising the fogging technique reported the highest prevalence estimates, followed by those that used non-cycloplegic refraction, while studies employing cycloplegic refraction reported the lowest prevalence. Comparable and substantial discrepancies were also observed between cycloplegic and non-cycloplegic measurements for myopia and other refractive errors. The refraction technique used is therefore a critical determinant of both the accurate assessment and classification of refractive status [43]. Evidence indicates that non-cycloplegic refraction tends to overestimate the prevalence of refractive errors, particularly in children aged 12 years or younger [44], and this effect may persist even in older age groups [45]. Although the precise mechanisms underlying these differences remain incompletely understood, cycloplegic refraction is widely regarded as the gold standard and is strongly recommended for the assessment of refractive status, especially in paediatric populations [44]. Accommodation can be controlled either pharmacologically or optically. Cycloplegia achieves this by inducing complete paralysis of the ciliary muscle, thereby eliminating accommodative responses. In contrast, optical fogging promotes accommodative relaxation through the use of plus lenses that introduce retinal defocus, such that any additional accommodation further degrades retinal image clarity [46]. Owing to this mechanism, fogging is generally more effective in older individuals with reduced accommodative amplitude, whereas cycloplegia is effective across all age groups. In the context of myopia diagnosis, cycloplegic refraction is considered essential, as the absence of cycloplegia introduces a systematic bias toward more myopic refractive outcomes [47]. Lin et al. demonstrated that optical fogging can effectively suppress unnecessary accommodative responses in young adults, significantly reducing myopic overestimation—particularly in individuals with marked accommodative overaction—although its efficacy remains inferior to that of cycloplegia [48]. Khan et al. reported that non-cycloplegic autorefraction yields more myopic refractive error estimates than cycloplegic autorefraction in young adults [49]. In contrast, the use of fogging lenses with open-field autorefractors for peripheral refraction in emmetropes and hyperopic individuals appears to achieve accommodative relaxation comparable to that induced by cycloplegia [49]. In the present systematic review, most included studies were conducted in school-based settings, where cycloplegic refraction is generally recommended [44]. However, only two of the 24 studies (9.52%) employed cycloplegia, raising concern that refractive error may have been systematically overestimated. Such overestimation has important implications, particularly for the accurate classification of refractive errors and subsequent management decisions.

As previously noted, the pooled prevalence of myopia in the present study was higher than that reported for sub-Saharan Africa [23]. This discrepancy may, in part, be explained by the relatively large proportion of included studies that did not employ cycloplegic refraction, which is known to overestimate myopia prevalence. Furthermore, substantial heterogeneity in myopia prevalence was observed across studies. For example, Abdul-Kabir et al. (2022) reported prevalence estimates ranging from as high as 40.0% (95% CI: 29.5–51.2) to as low as 0.5% (95% CI: 0.1–1.7). Such wide variability is likely attributable to differences in sample size, study populations, and refraction methodologies. These findings underscore the need for standardised and robust protocols for the assessment and management of myopia and other refractive errors in the country, particularly given their growing public-health significance. Nonetheless, when compared with reports from other global regions [21], myopia prevalence in Ghana remains relatively low and broadly comparable to estimates from other African settings [1, 23, 50].

A statistically significant association was identified between age group and the prevalence of overall refractive error. The prevalence was significantly lower in mid-childhood than in adolescence, yet higher than in adulthood. In contrast, the prevalence of hyperopia was significantly greater in mid-childhood compared with adolescence. This age-related reduction in hyperopia is consistent with the normal process of emmetropization, whereby physiological axial elongation during growth reduces hyperopic refractive states [2]. In addition, this pattern may reflect behavioural changes with age, including reduced outdoor activity and increased engagement in indoor and near-vision tasks, which are associated with a higher risk of myopia. Accordingly, the prevalence of myopia was significantly higher in adolescents than in children in mid-childhood (Fig. 3b), supporting this interpretation. A higher proportion of Ghanaian children aged 14 to17 years reportedly demonstrate increased use of smartphones for school-related activities, largely attributable to their affordability and functional reliability [51]. In contrast, children without access to smartphones commonly rely on internet cafés for academic purposes [51]. This pattern is consistent with the broader context of rapid economic growth and accelerating technological advancement observed across Africa, particularly in Ghana [26, 52]. Moreover, this trend may be associated with the well-documented progression of myopia during the school years [11], supporting the premise that age may serve as a useful predictor of myopia risk [53]. Although some studies report no significant age-related differences in myopia prevalence [23], others have demonstrated significant age-dependent variations [2, 40]. The observed positive association between myopia prevalence and increasing age underscores the need for age-specific myopia control strategies, as well as longitudinal studies to elucidate these patterns more clearly within the Ghanaian context. In addition, there is a critical need for policy-level interventions that promote early detection of refractive errors, particularly myopia, to facilitate timely and appropriate management. Such measures have the potential to mitigate or prevent adverse consequences on academic performance, future career opportunities, psychosocial development, and overall quality of life among affected children.

Regarding the study setting, myopia prevalence was significantly associated with the type of study site, with school-based studies reporting a significantly higher prevalence of myopia than community-based studies (Fig. 5b). Participants in school-based studies are typically younger and are more likely to be exposed to sustained near-work demands and prolonged indoor activities, both of which are established risk factors for the development of myopia. In contrast, community-based studies are more likely to include older individuals who engage in greater levels of outdoor activity and comparatively less near work, thereby conferring a relatively lower risk of myopia. In addition, pupils and students may experience increased exposure to digital devices for academic tasks, recreational activities, and screen-based entertainment, particularly in the context of recent advances in the integration of technology into the education system in Ghana. Enhancing Ghanaian student’s cognitive capacity and advanced skill sets in computer literacy and technological proficiency may facilitate effective communication across cultures, time zones, and geographical contexts, while equipping them for a rapidly evolving future [54]. However, this paradigm of increasing digitalisation concurrently exposes learners to vision-related risks, particularly myopia. Although the present findings indicate a higher prevalence of myopia in school-based studies, individuals outside formal school settings may not be insulated from this risk, as indoor activities and screen-based leisure time have increased substantially in the post-COVID-19 period [55, 56]. This underscores the need for targeted educational and awareness initiatives, alongside evidence-based myopia control and mitigation strategies. Consistent with these observations, other studies conducted in different regions of Africa have similarly reported higher prevalences of refractive errors in school-based populations compared with community-based samples [50].

Refractive errors were observed more frequently in females than in males, although the magnitude of this difference varied across subgroups. Sex-related differences in myopia prevalence have been attributed to an interplay of biological, behavioural, and sociocultural factors [57]. Evidence from Chinese adolescent populations indicates that a substantial proportion of the observed sex disparity in myopia may be explained by earlier pubertal onset in girls, which is associated with earlier and more rapid ocular growth [57]. Additionally, findings suggest that among females, earlier pubertal timing is linked to a faster progression of myopia compared with those who enter puberty later [58]. Furthermore, behavioural factors including greater engagement in near work activities, reduced time spent outdoors, and higher levels of smartphone use; have been reported more frequently among females than males [59, 60], potentially conferring an elevated risk for myopia onset and progression. These observations lend support to the consideration of sex-specific myopia prevention and control strategies, with particular attention to the pre-pubertal period, which may represent a sensitive or critical window of susceptibility among females. Within the Ghanaian context, however, additional research is required to clarify gender-related patterns of environmental exposure and their contribution to myopia risk, in order to inform and optimise context-appropriate myopia control interventions. While several studies [2, 53, 61] have documented a higher prevalence of myopia among females, others [55] report no significant sex-based differences in the distribution of refractive errors. Regardless of these inconsistencies, ensuring equitable access to refractive care services—including evidence-based myopia control interventions—remains essential for reducing visual impairment and preventing long-term ocular complications.

### Limitations and strengths of the study

The authors acknowledge several limitations that should be considered when interpreting the findings of this study. First, the review did not assess temporal trends in prevalence owing to the limited number of eligible studies, despite the absence of time restrictions in eligibility criteria. Second, substantial heterogeneity was observed in reporting practices across the included studies. Some studies did not report prevalence estimates for specific refractive error categories, while others stratified results by demographic variables such as age and sex. This variability reduced the number of studies eligible for inclusion in subgroup analyses.

Notwithstanding these limitations, the study has notable strengths. The systematic review was conducted within a clearly defined scope, employing a predefined search strategy and explicit eligibility criteria, and utilised multiple databases to ensure comprehensive identification of relevant studies. Study selection was initially undertaken by a single reviewer and subsequently validated by a second investigator, with any discrepancies resolved through adjudication by the research supervisors. In addition, data extraction and reporting adhered to established methodological standards, and the extracted data were subjected to rigorous and robust analytical procedures.

## Conclusion

The findings of this systematic review demonstrate that refractive errors, particularly myopia remain key public health concern in Ghana, and that prevalence estimates significantly varied by methodological and demographic factors. Reported prevalence estimates of myopia and other refractive errors were significantly higher in studies that did not employ cycloplegic refraction and in investigations conducted in school-based settings. With respect to age, hyperopia was more prevalent during mid-childhood, whereas the prevalence of myopia showed relative stability across age groups. In addition, prevalence rates of myopia, astigmatism and overall refractive error were consistently and significantly higher among females compared with males.

## Supplementary Information

Below is the link to the electronic supplementary material.

Supplementary Material 1

Supplementary Material 2

## Data Availability

No datasets were generated or analysed during the current study.
