# Supplementary material for: Systematic review and meta-analysis of refractive errors in Ghana with emphasis on myopia
Source: BMC Ophthalmol. 2026 Aug 3;26:706. doi: 10.1186/s12886-026-05179-9 (PMC13721436; doi:10.1186/s12886-026-05179-9)
Supplement: Supplementary file 1 [file 12886_2026_5179_MOESM2_ESM.docx]

**Figure S1.** Funnel plots and 95% Confidence Intervals (CIs) of refractive error.

**Figure S2.** Funnel plots and 95% Confidence Intervals (CIs) of myopia

**Figure S3.** Funnel plots and 95% Confidence Intervals (CIs) of hyperopia

**Figure S4.** Funnel plots and 95% Confidence Intervals (CIs) of astigmatism.

**Fig S1-S4** indicated the forest plot for refractive error, myopia, hyperopia and astigmatism. The points correspond to the standard error of the proportion from each study, and the dashed lines show the expected 95% confidence intervals around the summary estimate. All the studies were distributed symmetrically and fell within the 95% confidence limits which may suggests no substantial evidence of small-study effects or publication bias.

**Figure S5.** A meta-regression analysis of refractive error by year of publication.

**Figure S6.** A meta-regression analysis of myopia by year of publication.

**Figure S7.** A meta-regression analysis of hyperopia by year of publication.

**Figure S8.** A meta-regression analysis of astigmatism by year of publication.

**Fig S5-S8** indicated the meta-regression analysis of refractive error, myopia, hyperopia and astigmatism by year of publication. The vertical axis is the log proportion of refractive error, myopia, hyperopia and astigmatism, and the horizontal axis represents the year of publications. Each black dot represented one selected study, and the size of each black dot corresponds to the weight assigned to each study. Given that the slope of the regression line has descended slightly in this figure, this could be interpreted as the publication of the year indicating a weak strong association with the proportion of refractive error, myopia, hyperopia, and astigmatism. For every one-unit increase in refractive error, myopia, hyperopia and astigmatism decreases the publication year and but this association did not differ statistically (P > 0.05)
